# VEGF receptor targeted imaging of angiogenic response to limb ischemia in diabetic vs. non-diabetic Yucatan minipigs

**DOI:** 10.1186/s13550-020-00626-0

**Published:** 2020-05-12

**Authors:** Lynne L. Johnson, Jordan Johnson, Ziad Ali, Yared Tekabe, Rebecca Ober, Gail Geist, Alicia McLuckie, Aram Safarov, April Holland, Geping Zhang, Marina Backer, Joseph Backer

**Affiliations:** 1 Department of Medicine, Columbia University Medical Center, 622 West 168th St., PH 10-203, New York, NY 10032 USA; 2 Department of Veterinary Medicine, Columbia University, New York, NY USA; 3 Department of Pathology, Columbia University, New York, NY USA; 4 SibTech, Inc., Brookfield, CT USA

**Keywords:** Limb ischemia, Angiogenesis, Diabetes, VEGF, Yucatan minipigs

## Abstract

**Background:**

New therapies to treat diabetic peripheral artery disease (PAD) require target-specific non-invasive imaging modalities to follow efficacy. As a translational study, we performed targeted imaging of receptors for vascular endothelial growth factor (VEGF) in response to anterior femoral artery occlusion (FAO) in Yucatan minipigs and compare the normal response to response in diabetic Yucatan minipigs.

**Methods:**

Eleven Yucatan minipigs, 6 non-diabetic (non-D) and 5 purpose bred diabetic (D) (Sinclair, Auxvasse MO), underwent intravascular total occlusion of the anterior femoral artery (FA). At days 1 and 28, pigs underwent SPECT/CT ^201^Tl hindlimb perfusion imaging and at day 7 were injected with [^99m^Tc]DOTA-PEG-scVEGF (scV/Tc) tracer targeting VEGF receptor, and underwent biopsies of the hindlimb muscles for gamma counting and histology, followed by imaging. One day after the final scan, pigs underwent contrast angiography of the lower extremities. Counts from scans were converted to percentage injected activity (%IA).

**Results:**

Perfusion was lower in the occluded hindlimb compared to non-occluded on day 1 in both the D and non-D pigs. At day 7, scV/Tc count ratio of counts from ROIs drawn in proximal gastrocnemius muscle for the occluded over non-occluded limb was significantly higher in non-D vs. D pigs (1.32 ± 0.06 vs. 1.04 ± 0.13, *P* = 0.02) reflecting higher level of angiogenesis. Perfusion increased between days 1 and 28 in the muscles in the occluded limb for the non-diabetic pigs while the diabetic pig showed no increase (+ 0.13 ± 0.08 %IA vs. − 0.13 ± 0.11, *P* = 0.003). The anterior FA showed poor contrast filling beyond occluder and qualitatively fewer bridging collaterals compared to non-D pigs at 28 days.

**Conclusion:**

VEGF receptor targeted imaging showed the effects of diabetes to suppress angiogenesis in response to occlusion of the anterior femoral artery of purpose bred diabetic Yucatan minipigs and indicates potential applicability as a marker to follow efficacy of novel therapies to improve blood flow by stimulating angiogenesis in diabetic PAD.

## Introduction

The worldwide incidence of peripheral artery disease (PAD) is high and rising [1, 2]. Major contributing risk factors are age, smoking, hypertension, hyperlipidemia, and particularly diabetes. Symptomatic PAD is twice as common in diabetes, and the level of blood sugar has the most important association with PAD. For a 1% increase in HbA1c, the incidence of symptomatic PAD increases by 26% [3]. Current management guidelines include control of risk factors, anticoagulation, statins, and a few drugs such as cilostazol [4, 5]. Despite these management and treatment strategies, many patients have disease progression and require revascularization with limited success [6]. Finding new treatments and non-invasive tests to measure success are urgent clinical needs.

Diabetics have an attenuated angiogenic response to tissue hypoxia and poor collateral formation in response to vascular occlusion [7]. Vascular endothelial growth factor (VEGF) is the single most important stimulator of angiogenic response to hypoxia [8]. Therapies to increase VEGF locally are a therapeutic approach to improving blood flow in diabetic PAD. Imaging angiogenesis by targeting VEGF receptors would be a pathway-specific targeted approach to follow efficacy of such therapies.

Using non-diabetic murine hindlimb ischemia models of femoral artery ligation, investigators have targeted integrin to image angiogenesis with radiolabeled RGD probes [9–13]. An alternative target for molecular imaging is receptors for VEGF released locally in response to vascular occlusion [14, 15]. We previously compared [^99m^Tc]DOTA-PEG-scVEGF (scV/Tc) tracer targeting VEGF receptors 1 and 2 vs. ^99m^Tc-labeled RGD-based tracer, targeting α_v_β_3_ integrins in a mouse hindlimb ischemia model [16]. The radiotracer targeting VEGF receptors showed a more robust signal than the RGD tracer targeting α_v_β_3_. Immunofluorescense analysis indicated that differences in overall uptake of these two radiotracers in ischemic tissue can be explained by differences in affinity for the two probes to non-overlapping cell populations with uptake of scV/Tc seen on additional cell types [16]. The hindlimb vascular and muscle anatomy in a pig is closer to humans than in a mouse. As a translational model, we targeted VEGF receptors with scV/Tc in response to ligation of the anterior femoral artery in purpose bred diabetic Yucatan minipigs with poorly controlled blood glucose compared to normal pigs. To measure hindlimb perfusion responses to arterial ligation, we performed ^201^Tl imaging. Thallium perfusion imaging of the lower extremities or hindlimbs has been reported in patients with PAD and in pigs with arterial occlusions [17–19]. The aim of this study was to develop a new imaging strategy for clinical PAD to combine perfusion imaging and molecular targeting of VEGF receptors and test this approach to detect difference in angiogenic response to arterial occlusion in diabetic versus non-diabetic pigs.

## Methods

### Animals

All animal experiments were performed with the approval of the Institutional Animal Care and Use Committee of Columbia University. Purpose bred diabetic Yucatan minipigs (castrated male 27–30 kg) were obtained from Sinclair Laboratories (Auxvasse MO). The Sinclair diabetic Yucatan minipigs have type I diabetes induced with alloxan and are sent to investigators when diabetes is established and the blood sugar stabilized in range of 300–500 mg/dL on 7–8 units NPH Humulin N (range 2–12). When they arrived at Columbia University, each pig continued on the dose of insulin determined by Sinclair given once daily sub-cutaneous, and blood sugar monitored twice daily with a handheld glucometer (Accu-check Aviva, Roche) and additional doses of regular insulin given as needed. The veterinary staff weighed pigs weekly and observed them daily for signs of hyperglycemia or hypoglycemia. If any signs observed, the veterinarian was called, and pigs treated based on blood sugar levels and symptoms using protocols approved in our Institutional Animal Care and Use Committee (IACUC). None of the pigs in this study experienced any diabetic adverse event. Age and weight matched non-diabetic castrated juvenile male Yucatan minipigs were purchased from Sinclair Laboratory, Maine.

### Placement of endovascular occluder in anterior femoral artery

Veterinary staff sedated each pig with Telazol (2–5 mg/kg IM) and placed a catheter in an auricular vein. Propofol was given IV to facilitate intubation, and anesthesia was maintained with isoflurane inhalant for the duration of the procedure. The surgical site was prepared with betadine and alcohol alternated three times. A cut down was performed on the neck, the carotid artery isolated, and a pigtail catheter inserted over a guidewire into the artery and advanced to the iliofemoral trunk using small injections of contrast to identify catheter location (OEC digital fluoroscope model 7900) (General Electric). The pigtail was then replaced with an end hole catheter that was advanced to the left anterior FA just distal to the take-off of the profunda branch where an Amplatzer™ Vascular Plug II–8 mm (9-AVP2-008) (Abbott) was deployed into the catheter lumen. This plug is oversized compared to the vessel diameter and designed for vascular closure, acting as a barrier and becoming endothelialized. After deployment of the occluder, a contrast angiogram was performed to document the occlusion and site. The catheters were removed, the artery repaired, and the neck wound closed. The deep tissue layer was closed with 2-0 or 3-0 absorbable material in a simple continuous or interrupted pattern. The skin was closed with non-absorbable suture or stainless steel wound clips.

Pigs received peri-operative local analgesia with bupivacaine and antibiotic coverage with cefazolin. Post-procedure analgesia was provided with buprenorphine and carprofen, and antibiotic coverage was continued with Clavamox. At the end of each experiment (day 28), at the completion of contrast angiograms pigs were euthanized under anesthesia with an overdose of pentobarbital (Euthasol, IV).

### Radiotracer injection and hindlimb perfusion imaging

For all imaging procedures, pigs were sedated and catheters placed in auricular veins. Propofol was given to facilitate intubation, and sedated animals were placed in a HEPA-filtered cart for transport to the imaging facility. During transport, the pigs were given propofol as required to maintain adequate sedation and reconnected to inhalation anesthesia in the imaging room. Pigs were injected via ear vein catheter with 66.23 ± 9.62 MBq (1.79 ± 0.26 mCi) ^201^Tl day one after intravascular occlusion of the anterior femoral artery and 68.82 ± 7.03 MBq (1.86 ± 0.19 mCi) ^201^Tl on day 28 after FAO. The mean time between injection and imaging was 15.4 ± 10.8 min [19]. SPECT/CT imaging was performed on Philips Precedence camera (Philips, Andover MA) using the following parameters: for the SPECT scan ^201^Tl window (30% window for 72 keV photopeak, 20% window for 168 keV photopeak), 60° circular orbit (180° per head) for 64 steps, 40 s/step. For the LEGP collimator, the spatial resolution at 10 cm = 8 mm. Parameters for the spiral CT scan were the following: 120 KV, 3 mm slice thickness, 3 mm increments, and 25 mAs/slice.

### Angiogenesis imaging and muscle biopsies

On day 7 after vascular occlusion, pigs were sedated, intubated, and anesthetized as described above. ^99m^ Tc-labeled scV was prepared as previously described [16]. A dose of 296.37 ± 82.88 MBq (8.01 ± 2.24 mCi) of scV/Tc was injected through the ear vein catheter. The pigs remained anesthetized for approximately 3 h after injection to allow for blood pool clearance until transportation to imaging lab. Approximately 2 h after injection, hindlimb biopsies were obtained. The semimembranosus, biceps femoris, and gastrocnemius muscles were identified by palpation and biopsy sites in mid semimembranosus, mid biceps femoris, and proximal gastrocnemius identified, and hair was shaved over these sites. An incision approximately 1 cm in length was made with a scalpel, and subcutaneous tissue gently pushed aside down to muscle surface and using a 4 mm diameter bioptomes, 2 biopsies taken from each site on both the ischemic hindlimb and the opposite (control) hindlimb. The wounds were closed with sutures and dressed.

Pigs were transported to the Nuclear Cardiology Department and underwent SPECT/CT imaging on the Philips Precedence scanner using the following parameters: for the SPECT scan ^99m^Tc window (20% window for 140 KeV photopeak), 360° circular orbit (180° per head) for 64 steps, and 30 s/step. The CT parameters were the same as described above. The mean time between injection and imaging was 3.5 ± 0.33 h (range 3 to 4.25 h).

The wounds healed well without inflammation. Biopsy samples were weighed, counted in the gamma well counter (Wallac, 1470 Wizard), placed in formalin followed by alcohol, embedded, and sectioned for histology.

### Contrast angiogram and necropsy

On the day following the final thallium scan, pigs underwent a contrast angiogram of the hindlimb circulation. Following sedation, intubation, and under general anesthesia using sterile conditions, a cut-down was performed on the right carotid artery and a 5 French pigtail catheter advanced to the distal aorta just proximal to the iliac bifurcation. Iodinated contrast (678 mg Optiray 320) was selectively injected into each common femoral artery, and images obtained to include the anterior FA proximal and distal to the occluder and the profunda branch. Following this final angiogram, anesthesia was deepened, and pigs euthanized with Euthasol (100 mg/kg IV) by veterinary staff. At necropsy, approximately one to two 3–4 cm^3^ samples of muscle were taken from the proximal and distal gastrocnemius, biceps femoris, semimembranosus, and semitendinosus for both hindlimbs. Two 1 cm^3^ samples were taken from each of these larger sections for gamma well counting.

### Scan analysis

**Fig. 1:**
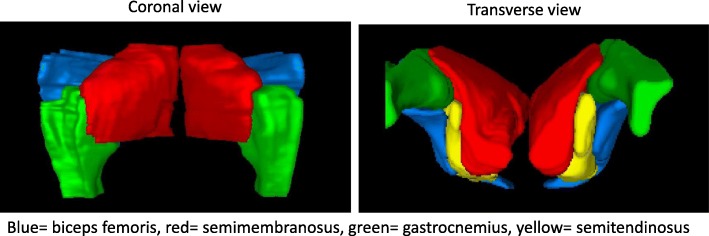
Coronal and transverse 3D rendering of the volumetric pig hindlimb muscles from the CT images used to identify muscle boundaries to measure ^201^Tl uptake for each muscle. Color code explained below images.

DICOM files for the ^201^Tl scans including the attenuation corrected (AC) SPECT/CT images of hindlimbs were uploaded onto the MIM 64-bit software (MIM Software, Cleveland, OH). Using the CT image, hindlimb muscle groups including the gluteus (Glut), semimembranosus (SM), semitendinosus (ST), bicep femoris (BF), and gastrocnemius (Gas) were analyzed (Fig. 1). Using operator guided identification of fiduciary points and computer assisted boundary definition, 3D contours of each muscle were determined using the CT scan as guide for segmentation. SPECT scan data was superimposed and carefully aligned with the CT scan data. Using the statistics function, total counts and volumes in cc were recorded for each muscle. Counts were converted to percentage injected activity (IA) with conversion factors derived from phantom experiments in which 1 mCi of either ^201^Tl or ^99m^Tc were placed in I cc Eppendorf tube suspended in middle of a phantom, and scan acquired using same protocol as the pig scans.

For the scV/Tc scans, counts were obtained for regions drawn around the proximal gastrocnemius for the occluded and non-occluded limbs and expressed as ratios.

### Contrast angiogram analysis

Images were obtained from OEC digital fluoroscope model 7900 (General Electric) and uploaded onto a computer running the ImageJ software. Segments of the anterior FA distal to the occluder and the profunda artery with similar backgrounds were selected, and a region of interest (ROI) of the same size was placed over the two vessels. Using the measuring tool, mean value for contrast intensity was measured for the two vessel regions, and ratios determined for the occluded segment over the normal artery for both the non-diabetic and diabetic animals.

### Histology

All tissue samples from biopsies and necropsy were placed in formalin for 48 h followed by 70% alcohol and then embedded. For immunohistochemical analyses, serial sections were deparaffinized in xylene, treated with 0.3% hydrogen peroxide for 20 min, and incubated in protein-free block (Dako Inc., Carpinteria, CA, USA) for 10 min to inhibit the non-specific binding of primary antibody. All sections were stained with hematoxylin and eosin (H&E). Staining for capillary sprouting was performed using biotinylated Griffonia Bandeiraea Simplicifolia Isolectin I (1:50; Vector Laboratories, Burlingame, CA) and treated for 30 min with VECTASTAIN ABC reagent (Vector Laboratories), followed by 3′,3′-diaminobenzidine (DAB substrate kit for peroxidase; Vector Laboratories, Burlingame, CA, USA), and counterstaining with Gill’s hematoxylin solution. Morphometric and immunohistochemical analyses of the arterial segments were performed using a Nikon microscope (Tokyo, Japan) and the Image-Pro Plus software (Media Cybernetics Inc., Silver Spring, MD, USA).

### Statistical analysis

Comparisons between groups for all variables were made using 2 sample *t* test to compare unpaired data between groups with a *P* value of < 0.05 to indicate statistical significance. Values for %IA ^201^Tl uptake for individual hindlimb muscles on the final scan were correlated with anatomically corresponding sections from muscles excised during necropsy measured in the gamma well counter (%IA/g) using the Pearson product-moment correlation coefficient.

## Results

### Animals

The average age of the pigs on arrival was 8.9 ± 0.3 months of age. The average weight at the end of the study was 30.6 ± 3.9 kg for the non-diabetic pigs and 31.3 ± 4.1 kg for the diabetic pigs. For the diabetic pigs, blood glucose levels ranged from 183 ± 95 to 590 ± 108 mg/dL. The average morning insulin dose was 2.4 ± 0.5 units, and afternoon insulin dose was 3.3 ± 1.5 units to keep levels within the protocol limits.

### Intravascular occlusion

The occluders were successfully deployed in the lumen of the anterior femoral artery just distal to the take-off of the circumflex branch in all pigs. There was no contrast seen in the vessel distal to the occluder.

### Hindlimb perfusion at day 1 and day 28 after FAO

**Fig. 2:**
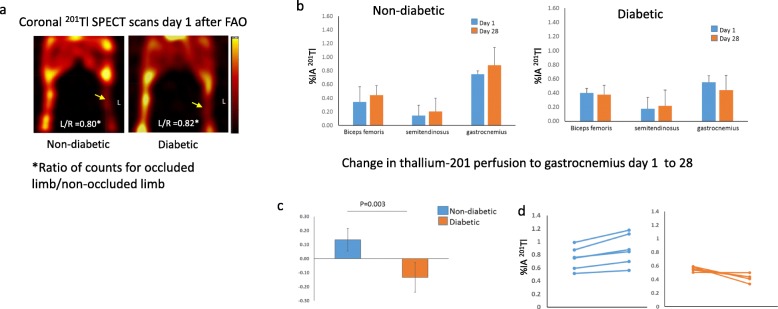
**a** Coronal ^201^Tl SPECT images of non-diabetic and diabetic pig imaged 24 h after L FAO showing reduced tracer uptake in the occluded limb (yellow arrows) compared to the non-occluded limb. The color scale bar represents counts/unit time. Mean values of ratios of total hindlimb counts for the ischemic/non-ischemic (L/R) limbs for the non-diabetic and diabetic pigs are shown on the images. **b** Bar graphs of mean ± SD ^201^Tl uptake as %IA for the three distal hindlimb muscles for day 1 and day 28 scans for non-diabetic group (left graph) and diabetic group (right graph). **c** Mean ± SD for the difference in ^201^Tl uptake (%IA) in the gastrocnemius between the 2 time points for the two groups. The difference was significant (*P* = 0.003). **d** Individual values for ^201^Tl uptake (%IA) at day 1 and day 28 for the gastrocnemius muscle for non-diabetic pig (blue lines) and diabetic pig (orange lines)

Visually, there appeared to be less uptake of ^201^Tl in the occluded left hindlimb compared to the non-occluded right hindlimb (Fig. 2a). Ratio of counts in the occluded leg over the non-occluded leg for both diabetic and non-diabetic pigs was less than 1.0. We propose several explanations for the small difference in perfusion between the two hindlimbs in the “Discussion” section.

The hindlimb volumetric muscle boundary map for measuring individual muscle values for count determination is shown in Fig. 1. Perfusion in the occluded hindlimb muscles tended to be higher at day 28 compared to day 1 for the non-diabetic pigs compared to the diabetic pigs (Fig. 2a). Mean value for perfusion in the gastrocnemius muscles in the occluded hindlimb in the non-diabetic pigs improved from day 1 to day 28 (+ 0.13 ± 0.08) (range 0.04–0.24 %IA) compared to the diabetic pigs where perfusion was unchanged or lower (− 0.13 ± 0.11) (range − 0.25 to 0.00 %IA) (Fig. 2b). Changes in gastrocnemius perfusion for individual animals are shown in Fig. 2c.

Biopsy samples taken from all of the hindlimb muscles at necropsy were counted for ^201^TI activity in the gamma well counter. The values for %IA from the scan correlated significantly with %IA/g on gamma well counting (*R*^2^ = 0.5619, *P* < 0.001).

### Angiogenesis at day 7 after FAO

**Fig. 3:**
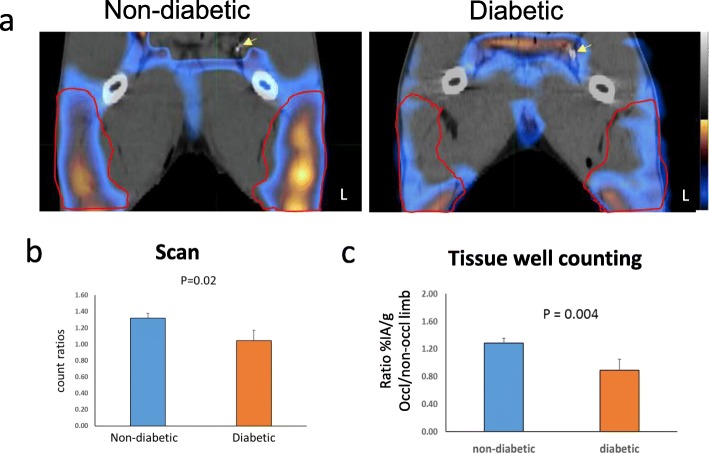
**a** scV/Tc SPECT/CT scans from day 7 after FAO for a non-diabetic pig on the left and a diabetic pig on the right. Yellow arrows identify the occluder on the CT scan. Red outlines show borders of the proximal gastrocnemius muscle identified on the CT. The color scale bar represents counts/unit time. The bladder was removed, and the upper threshold reduced equally in both images to about 50% of peak counts to visually bring out the muscle uptake. The raw counts derived from the images were moved to spread sheet to convert to %IA (see text). **b** Bar graphs of mean ± SD of ratios of tracer uptake (%IA/cc) between the two limbs taken from gastrocnemius regions. **c** Bar graph of ratios for %IA/g between the two limbs measured from semimembranosus biopsy specimens taken immediately prior to scans

**Fig. 4:**
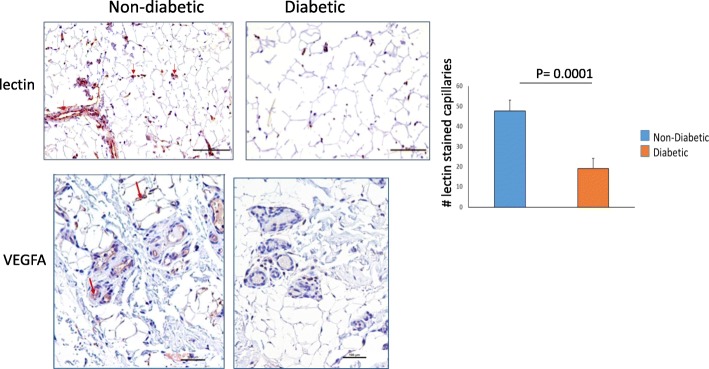
Immunohistochemical staining on muscle biopsy samples taken at day 7 after FAO. Lectin stained sections below show more staining for lectin (brown chromogen) for the non-diabetic pig compared to the diabetic pig (magnification × 200). The bar graph on the right shows mean ± SD for quantification of the lectin stained capillaries for all biopsy specimens in the two groups of pigs. The non-diabetic pigs had significantly higher lectin staining. The second set of sections shows greater staining for VEGFA (brown chromogen) in the non-diabetic compared to diabetic pig

Focal uptake of scV/Tc in muscles distal to the occluder was seen on all scans for the non-diabetic pigs. The diabetic pigs showed no focal uptake of the tracer distal to the occluder (Fig. 3a). Percentage IA of scV/Tc uptake from volumes drawn for the proximal gastrocnemius for both limbs were expressed as ratios occluded/non-occluded limb. The mean ratio for the diabetic pigs was 1.04 ± 0.13 (range 0.90–1.21) while the ratio for the non-diabetic pigs was 1.32 ± 0.06 (range 1.25–1.36) (*P* = 0.02) (Fig. 3b). These findings were corroborated by the gamma well counting of the biopsy samples taken from the muscles just prior to imaging (Fig. 3c). Quantitative immunohistology for lectin on biopsy specimens showed greater lectin staining (expressed as number of capillaries per field) indicating greater capillary sprouting in the non-diabetic pigs (47 ± 6) (range 38–55) vs. diabetic pigs (19 ± 5) (range 14–26), *P* = 0.0001 (Fig. 4). Staining for VEGFA was qualitatively higher in non-diabetic pig biopsy samples (Fig. 4).

### Collaterals

**Fig. 5:**
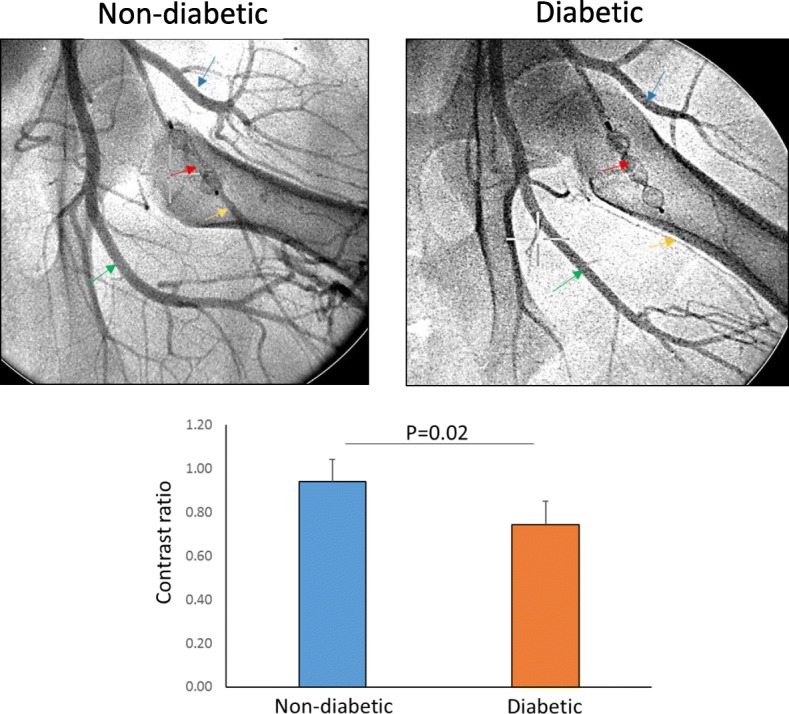
Top images show contrast angiograms performed at day 28 after FAO immediately prior to sacrifice showing the occluder (red arrow) and the anterior femoral artery immediately distal to the occluder (yellow arrow). The non-diabetic pig (left) shows good contrast opacification of the vessel immediately distal to the occluder while the diabetic pig (right) shows poor opacification. The green arrow identifies the profunda artery and blue arrow the circumflex branch of the anterior femoral artery that are providing more abundant collaterals in the non-diabetic pig than the diabetic pig. The bar graph below shows mean ± SD for ratios of contrast intensity between the distal anterior femoral artery and the profunda artery for the non-diabetic and diabetic pigs

Contrast angiograms showed reconstitution of the anterior FA back to the distal occluder from collaterals coming from the circumflex branch and the profunda artery in all the non-diabetic pigs while the diabetic pigs showed poor reconstitution of the distal anterior FA and qualitatively fewer collaterals from the circumflex and profunda. The ratios of contrast intensities between the distal anterior FA and profunda were 0.94 ± 0.10 (range 0.90–1.09) for non-diabetic pigs and 0.74 ± 0.11 (range 0.56–0.83) for the diabetic pigs (*P* = 0.02) (Fig. 5).

## Discussion

The prevalence of diabetes and of diabetic peripheral artery disease (PAD) continues to rise in the USA and worldwide. Severity of PAD is classified based on symptoms using the Rutherford classification of I–VI [6]. Patients with classes I–III have reduced exercise tolerance due to ischemic leg pain (claudication). Patients with more advanced disease (classes IV–VI) have chronic limb ischemia (CLI) with pain at rest, tissue loss, and gangrene. More advanced disease occurs most frequently in diabetics and is associated with diffuse vascular disease and occlusions. Some cell-based targeted therapies attempt to improve limb perfusion by improving angiogenesis in hypoxic tissue [20]. The results of this translational model of diabetic vascular disease suggest that a radiolabeled probe targeting VEGF receptor expression may be useful to document therapeutic response.

VEGF is the single most important regulator of angiogenesis [8, 21–23]. Secreted by endothelial cells, smooth muscle cells, and myocytes, members of the VEGF families bind to one or more of three tyrosine receptors: VEGFR-1 (flt1), VEGFR-2 (flk1), and VEGFR-3 (22). The human VEGF-A gene through alternate exon splicing generates 4 isoforms with different numbers of amino acids: VEGF121, VEGF165, VEGF189, and VEGF206. Coordinated activation of VEGFR-1 and VEGFR-2 plays an important role to stimulate new vessel formation, restore muscle perfusion, and promote muscle regeneration in PAD [24–26]. The radiotracer we used to image angiogenesis, [^99m^Tc]DOTA-PEG-scVEGF, is based on an engineered single-chain (sc) recombinant version of VEGF121, site-specifically derivatized with PEGylated chelator DOTA for radiolabeling with ^99m^Tc (14,15). scV/Tc and its receptor-selective versions show nanomolar affinity to VEGFR-1 and VEGFR-2 is readily internalized upon binding to the VEGF receptors, and it reliably detects VEGF receptor expression in murine models of atherosclerosis and angiogenesis [16, 27].

This is the first study to show feasibility of molecularly targeting angiogenesis in response to vascular occlusion in a diabetic large animal model. Investigators have used mice extensively as a model of hindlimb ischemia to evaluate radiotracers targeting angiogenesis including diabetic mice (alloxan induced) [27]. The muscle anatomy of the mouse hindlimb is different from a large animal or human. In the murine model of hindlimb ischemia, the femoral artery is ligated, and the entire tibialis muscle which is a large muscle in the mouse shows hypoxic damage. Despite this injury, the mouse continues to walk on all limbs. At 5 days after ligation, peak time for angiogenesis in the mouse, there is extensive tracer uptake in the mouse hindlimb which translates to improved blood flow by ultrasound imaging (US) at day 22 [9–11] and extensive increase in vascularity in tibialis muscle as shown on micro-CT by Kapanadze et al. [11]. The vascular anatomy of the pig is closer to human than is the mouse anatomy. The major arteries supplying the distal hindlimb in the pig are the anterior femoral and profunda arteries. Occluding the common femoral or iliac in the pig would lead to severe limb ischemia and inability to walk. By occluding the anterior femoral artery distal to the circumflex branch, posterior perfusion is maintained via the profunda artery. In the normal pig, focal uptake of scV/Tc and angiogenesis was localized to the proximal gastrocnemius. In the diabetic pigs, there was no focal uptake seen on scans, and quantitative uptake ratios for the two limbs were 1.0. A major mechanism for failure of angiogenesis in response to local tissue hypoxia in diabetics is attributed to the effect of Receptor for Advanced Glycated Endproducts (RAGE) which is highly expressed in the vasculature of diabetics and blocks angiogenesis [28, 29, 30]. We have previously shown that the normal angiogenic response to FAL in WT diabetic mice is restored in RAGE null diabetic mice [31] and in diabetic mice treated with an antibody blocking RAGE [32].

Both angiogenesis and arteriogenesis (collateral formation) contribute to re-establish flow beyond an arterial occlusion [33]. Angiogenesis is a dynamic process at the molecular level initiated by hypoxic stimulus and involving remodeling of the extracellular space and endothelial cell proliferation and formation of microvessels. The stimulus for arteriogenesis involves local physical factors beginning with increased sheer stress to pre-existing collateral vessels. This physical force stimulates expression of adhesion molecules, cytokines, and growth factors by the endothelium leading to outward remodeling of the vessel wall to form a collateral artery [34]. The diabetic pigs in this study had reduced arteriogenesis in addition to angiogenesis in response to arterial occlusion as shown by poorer collateral formation around the site of occlusion. The factors causing the failure of diabetics to respond to local physical forces causing outward remodeling of collateral vessels are not known.

The overall perfusion to the gastrocnemius fell from day 1 to day 28 for the diabetic pigs. Individual responses either showed to change for a fall. We attribute this abnormal response to combination of failure of angiogenesis, poor collateral formation, and progression of diffuse vascular disease.

## Conclusions

In summary, the results of this study document that the purpose bred diabetic Yucatan minipig mimics several of the important characteristics of diabetic vascular disease in humans including suppression of angiogenesis. These observations suggest that VEGF receptor imaging shows promise as a molecular targeted imaging approach to follow success/failure of therapies to improve angiogenesis in PAD.

## Limitations

The pig model we chose to study (purpose bred diabetic Yucatan minipig) mimics type I diabetes while type II diabetes is the most common in humans. We chose this model because of the consistent hyperglycemia in range of poorly controlled human diabetes that is associated with development of diabetic complications including PAD. Porcine models of metabolic syndrome do not consistently have hyperglycemia [33]. Further refinements of this model such as high fat diet and longer follow-up after FAO to generate atherosclerosis would better mimic human disease.

There was only a small reduction in perfusion in the occluded limb compared to the non-occluded limb on the first thallium scan performed 24 h after FAO. Perfusion in the occluded limb for this first time point may have been lower if we had imaged earlier after the occlusion. Twenty-four hours between injection and imaging allowed blood flow to increase to the occluded limb from the profunda artery. In addition to delayed imaging, increases in vascular permeability associated with early angiogenesis would hasten uptake of ^201^Tl into the myocellular space.

There is concern that the process of taking biopsy samples at day 7 before the scV/Tc scan may have contributed to uptake of tracer. Any surgical intervention increases the angiogenic signal due to effect of wound healing. We were careful in the process of taking hindlimb muscle biopsies to minimize tissue injury. Any uptake of tracer related to the biopsies would appear as small focal hotspots on the muscle surface on the SPECT scans and were not seen.

## Data Availability

The datasets used and/or analyzed during the current study are available from the corresponding author on reasonable request.

## Abbreviations

CT: Computerized tomography
DOTA: Dodecane tetra acetic acid
FA: Femoral artery
FAO: Femoral artery occlusion
PAD: Peripheral artery disease
PEG: Polyethylene glycol
RAGE: Receptor for Advanced Glycated Endproducts
SPECT: Single Photon Emission Computerized Tomography
VEGF: Vascular endothelial growth factor
